# Low pH Environmental Stress Inhibits LPS and LTA-Stimulated Proinflammatory Cytokine Production in Rat Alveolar Macrophages

**DOI:** 10.1155/2013/742184

**Published:** 2013-10-30

**Authors:** Stanley F. Fernandez, Christopher Fung, Jadwiga D. Helinski, Ravi Alluri, Bruce A. Davidson, Paul R. Knight

**Affiliations:** ^1^Department of Medicine, Division of Cardiovascular Medicine, University at Buffalo, Suite 7030, 875 Ellicott Street, Buffalo, NY 14203, USA; ^2^Department of Anesthesiology, University at Buffalo, 3435 Main Street, Buffalo, NY 14214, USA; ^3^Department of Microbiology, University at Buffalo, 3435 Main Street, Buffalo, NY 14214, USA

## Abstract

Gastric aspiration increases the risks for developing secondary bacterial pneumonia. Cytokine elaboration through pathogen recognition receptors (PRRs) is an important mechanism in initiating innate immune host response. Effects of low pH stress, a critical component of aspiration pathogenesis, on the PRR pathways were examined, specifically toll-like receptor-2 (TLR2) and TLR4, using isolated rat alveolar macrophages (aMØs). We assessed the ability of aMØs after brief exposure to acidified saline to elaborate proinflammatory cytokines in response to lipopolysaccharide (LPS) and lipoteichoic acid (LTA) stimulation, known ligands of TLR4 and TLR2, respectively. Low pH stress reduced LPS- and LTA-mediated cytokine release (CINC-1, MIP-2, TNF-*α*, MCP-1, and IFN-*β*). LPS and LTA increased intracellular Ca^2+^ concentrations while Ca^2+^ chelation by BAPTA decreased LPS- and LTA-mediated cytokine responses. BAPTA blocked the effects of low pH stress on most of LPS-stimulated cytokines but not of LTA-stimulated responses. *In vivo* mouse model demonstrates suppressed *E. coli* and *S. pneumoniae* clearance following acid aspiration. In conclusion, low pH stress inhibits antibacterial cytokine response of aMØs due to impaired TLR2 (MyD88 pathway) and TLR4 signaling (MyD88 and TRIF pathways). The role of Ca^2+^ in low pH stress-induced signaling is complex but appears to be distinct between LPS- and LTA-mediated responses.

## 1. Introduction

Gastric aspiration pneumonitis is a major cause of death and disability. An important complication of this acute lung injury is the development of a secondary pulmonary bacterial pneumonia from inhalation of oropharyngeal or gastric bacterial flora. Patients that are specifically susceptible to gastric aspiration pneumonitis are those with impaired airway defense reflexes such as anesthetized patients or patients with altered mental status. Consequences of low pH gastric aspiration can range from benign aseptic pneumonitis to acute respiratory distress syndrome (ARDS) with or without a fulminant secondary bacterial infection. It is estimated that complications from gastric aspirations can be seen in 1 of every 2,000–4,000 anesthetic cases every year and possibly 10–15% of all community acquired pneumonia [1, 2].

Common bacterial organisms implicated in aspiration pneumonia include *Streptococcus pneumoniae, Staphylococcus aureus, Haemophilus influenzae*, and Enterobacteriaceae [1]. Although gastric components are highly variable with varying proportions of gastric particles, acidity, and bacterial load, it appears that the extent of pulmonary injury is directly related to the aspirate volume, acidity, and composition [3, 4]. In rat models of gastric aspiration injury, intratracheal administration of acidified solution (pH of 1.25) results in an early direct corrosive irritation to the airway epithelium followed by a delayed inflammatory response (4–6 hours after exposure) characterized by neutrophil activation and extravasation into the air spaces and elaboration of proinflammatory mediators and chemotactic cytokines [3, 5, 6]. Gastric aspiration is an important risk factor for bacterial pneumonia [7]. Using an *in vivo* rat model, our laboratory has demonstrated that a preceding gastric aspiration injury results in reduced clearance of *Escherichia coli* instilled into the lungs [7]. The mechanism by which transient pulmonary low pH stress promotes bacterial infection is the focus of this investigation.

Our major hypothesis is that a transient low pH environment resulting from gastric aspiration adversely affects alveolar macrophage (aMØ) antimicrobial function. Using isolated rat aMØs, we investigated the effects of a transient low pH exposure in impairing the ability of these cells to express cytokines in response to ligation of pathogen recognition receptors (PRRs). Macrophages were stimulated, *in vitro*, with the pathogen associated molecular pattern (PAMP) ligands of *E. coli* lipopolysaccharide (LPS) or pneumococcal lipoteichoic acid (LTA), known ligands of toll-like receptor (TLR) 4 and TLR2, respectively. TLRs are PRRs utilized by immune sentinel cells to recognize microbial PAMPs to illicit antibacterial proinflammatory responses [8]. The aMØs' response to receptor ligation was quantified based on protein levels of cytokine-induced neutrophil chemoattractant-1 (CINC-1), macrophage inflammatory protein-2 (MIP-2), tumor necrosis factor-*α* (TNF-*α*), macrophage chemoattractant protein-1 (MCP-1), and interferon-*β* (IFN-*β*) in the culture supernatant, as assessed by ELISA. The role of cytosolic Ca^2+^ in mediating the effects of low pH stress was also investigated [9, 10] using the Ca^2+^-sensitive dye, Fura-2, in single cell imaging studies or the Ca^2+^ chelator, BAPTA, in cell culture stimulation studies. These findings will help identify the mechanisms by which gastric aspiration can alter MØ function and modify innate immune responses that may predispose patients to secondary pulmonary bacterial infections.

## 2. Materials and Methods

### 2.1. Rat Alveolar Macrophage Isolation

Bronchoalveolar lavage (BAL) was performed on naïve Long-Evans rats (Harlan Sprague-Dawley, Indianapolis, IN) weighing 250–390 g to isolate alveolar macrophages for *in vitro* experiments. Briefly, rats were anesthetized with 2-3% halothane in 100% O_2_ to full anesthetic effect and then exsanguinated by transecting the inferior vena cava. After thoracotomy, the thoracic and pulmonary vasculatures were flushed using 20 mL of Hank's balanced salt solution with Ca^2+^ and Mg^2+^ (HBSS) injected directly into the right ventricle. A 14-gauge catheter was inserted and secured in the trachea and BAL performed with 50 mL of normal saline instilled in 10 mL aliquots. The instilled saline solution was recovered and kept in ice until processing.

### 2.2. Cell Plating

The recovered BAL fluid was centrifuged at 1,500 ×g for 3 min at 4°C and the cell-free supernatant discarded. The cell pellet was resuspended in 4 mL of culture media (RPMI-1640, Life Technologies, Carlsbad, CA), containing 10% heat-inactivated fetal calf serum and penicillin and streptomycin. A cytoslide was prepared with 5 × 10^4^ cells plated onto a slide and centrifuged at 28 ×g (Cytospin 3 cytocentrifuge, Shandon, Pittsburgh, PA) for 5 min, air dried, and stained with Diff-Quik (Dade Behring, Newark, DE). Microscopic assessment demonstrated that plated cells were ≥98% macrophages. Alveolar macrophages were >95% viable prior to plating as determined by Trypan Blue exclusion. Cells were resuspended in warm culture media and seeded in poly-L-lysine-coated 24-well polystyrene tissue culture plates at a density of 2 × 10^5^ cells/well, 500 *μ*L/well for ELISA studies, or 200 *μ*L/well in 96-well plates for WST-1 cell viability (Roche Diagnostics, Indianapolis, IN) studies. Cells were incubated at 37°C, 95% RH, and 5% CO_2_ (all incubations were performed under these conditions unless otherwise noted) for 20–22 hrs to allow cells to attach and quiesce.

### 2.3. Treatment Conditions

Plated cells were washed with an unbuffered balanced salt solution (UBSS, 144 mM NaCl, 5.4 mM KCl, 1.8 mM CaCl_2_, and 0.8 mM MgCl_2_); then 100 *μ*L/well of appropriate injury solution, UBSS (uninjured control) or UBSS + 1 N HCl, pH = 1.75 (acidic injury), was added to each well. After 1 min of injury exposure, 400 *μ*L of culture media was added (neutralizing the injury solution) and then removed and replaced with 500 *μ*L/well fresh culture media containing 1.5 *μ*g/mL LPS, 30 *μ*g/mL LTA, or media alone (nonstimulated control). The cultures were incubated, as above, for 24 hrs and the supernatant collected and stored at −80°C for subsequent analysis. A pH = 1.75 was specifically chosen to represent the most acidic injury that did not cause significant reduction in macrophage viability. This was based on a study examining a 1 min exposure to a range of pH from 1.25 to 3.00 using a WST-1 cell viability assay (Roche Diagnostics, Indianapolis, IN), a colorimetric measure of cellular metabolic activity. Incubation with pH 1.75 for 1 min was established as the most appropriate pH to maximize low pH injury without producing cell death. For intracellular calcium sequestration experiments, macrophages were incubated with 5 *μ*M BAPTA-AM (an intracellular Ca^2+^ chelator) in culture media (Life Technologies, Grand Island, NY) for 30 min prior to initiating the acidic injury and LPS or LTA stimulation procedures.

### 2.4. Cytokine Analysis

Cell-free culture media were analyzed by ELISA to determine concentrations of CINC-1, MIP-2, and MCP-1 using their respective antibody pairs and recombinant protein standards from R&D Systems (Minneapolis, MN) and IFN-*β* from USCN Life Sciences (Houston, TX), as previously described [11]. TNF-*α* levels were assessed by cytotoxicity bioassay using WEHI 164, subclone 13 cells (a generous gift from Dr. Steven L. Kunkel, Department of Michigan, Ann Arbor, MI). This cell line is a TNF-*α*-sensitive line derived from a mouse fibrosarcoma which allows assessment of bioactive TNF-*α* as previously described [11].

### 2.5. Fluorescence Calcium Imaging

Freshly isolated alveolar macrophages (2 × 10^5^ cells in 200 *μ*L culture media) were dispensed into the 10 mm diameter microwell in the middle of a 35 mm diameter glass bottom plastic culture dish with a poly-L-lysine-coated no. 1.5 glass coverslip defining the well bottom (MatTek Corp., Ashland, MA). The cells were incubated for 2 hrs to allow cell attachment. An additional 2 mL of culture medium was added, and the cells were incubated for 20–22 hrs. The *in vitro* injury procedure described above (UBSS + HCl, pH = 1.75 for 1 min) was performed, the cells washed, and culture media containing 1.5 *μ*g/mL LPS or 30 *μ*g/mL LTA added. Following the appropriate duration of incubation, the cells were washed 2x with Buffer A (124 mM NaCl, 5.8 mM KCl, 1.4 mM KH_2_PO_4_, 18.7 mM HEPES, 14.1 mM dextrose, 0.68 mM mannitol, and 1.8 mM CaCl_2_, pH = 7.4) and then incubated with 6 *μ*g/mL Fura-2 AM (Life Technologies, Grand Island, NY) for 30 min. The cells were then washed twice with Buffer A and incubated for an additional 15 min in Buffer A to allow the Fura-2 AM to deesterfy. The culture dish was placed on an inverted microscope stage of a dual-excitation microscopic fluorescence spectrophotometer (Photon Technology International, Birmingham, NJ) that was used to excite the dye at 340 and 380 nm, alternating at a 2 Hz sampling rate. Fluorescence emissions at 510 nm were recorded as an excitation fluorescence ratio (*R*_340/380_, which is directly proportional to the intracellular calcium concentration, [Ca^2+^]_i_) by a digital PMT detector that was masked to allow emission measurements from a single alveolar macrophage. Measurements were taken for one minute on 10 different cells for each time point. For the immediate and 30 min time points, the cells were loaded with Fura-2 AM prior to low pH and LPS or LTA exposure.

### 2.6. Mouse Acid Aspiration Injury Model

Eight-week-old CD-1 male mice (Charles River Laboratories, Wilmington, MA) were used for *in vivo* experiments to determine the effects of low pH injury on bacterial clearance. After induction of halothane anesthesia, a tracheotomy was performed by midline incision and blunt dissection. A 22 ga needle was inserted into the trachea under direct visualization, and 3.6 mL/kg normal saline, pH = 5.3 (NS), or NS + HCl, pH = 1.25, (Acid) was injected followed by a 0.2 mL bolus of air while the mouse was suspended by its incisors in a 60° supine position. The bacterial challenge inoculum, 50 *μ*L, was instilled through the same needle 1 min following the NS or Acid instillation. Two separate bacterial inocula were prepared, *Escherichia coli* (*E. coli*, CP9, an extraintestinal pathogenic *E. coli* strain) and *Streptococcus pneumoniae* (*S. pneumoniae*, strain: EF3030). *E. coli* was prepared by culturing overnight at 37°C in Luria-Bertani medium and centrifuged at 8,000 ×g for 4 min at 4°C and then resuspending the pellet in sterile NS to 5 × 10^6^ colony-forming units (cfu)/50 *μ*L. *S. pneumonia* cultures were prepared from frozen stocks of known titer that were centrifuged as above and the pellet resuspended in sterile NS to 5 × 10^5^ cfu/50 *μ*L. Actual inocula titers were determined by serial titration just prior to each *in vivo* injury experiment. Mice were sacrificed at 24 hrs after intratracheal challenge by exsanguination during halothane anesthesia. Lungs were removed and homogenized by adding sterile lung homogenate buffer, pH = 7.4 (150 mM NaCl, 15 mM Tris base, 1 mM CaCl_2_·2H_2_O, 1 mM MgCl_2_·6H_2_O containing 500 *μ*M AEBSF HCl, 150 nM aprotinin, 1 *μ*M E-64, 0.5 mM disodium EDTA, and 1 *μ*M leupeptin hemisulfate) (protease inhibitor cocktail set I, Calbiochem/EMD Chemicals, Gibbstown, NJ), such that the total lung plus buffer weight was 3 g (i.e., 3 mL) and homogenizing on ice with a Polytron PT-2000 tissue homogenizer (Brinkman Instruments, Westbury, NY). Bacterial titer (cfu/mL) of the homogenized tissue was determined by serial titration and the bacterial load determined by multiplying the lung homogenate titer by the total volume (i.e., 3 mL). Bacterial clearance was expressed as log_10_(starting inoculum/lung homogenate bacterial load at time of harvest). All procedures performed on animals were approved by the Institutional Animal Care and Use Committee at the University at Buffalo and complied with all state, federal, and National Institutes of Health regulations.

### 2.7. Statistical Analysis

All data are expressed as mean ± SEM. Statistical analyses were performed using one-way ANOVA for multiple group analysis and two-tailed Student's *t*-test for direct group comparisons with *P* < 0.05 considered as significant.

## 3. Results

### 3.1. Effects of Low pH Stress on LPS-Induced Cytokine Production in Alveolar Macrophages

**Figure 1:**
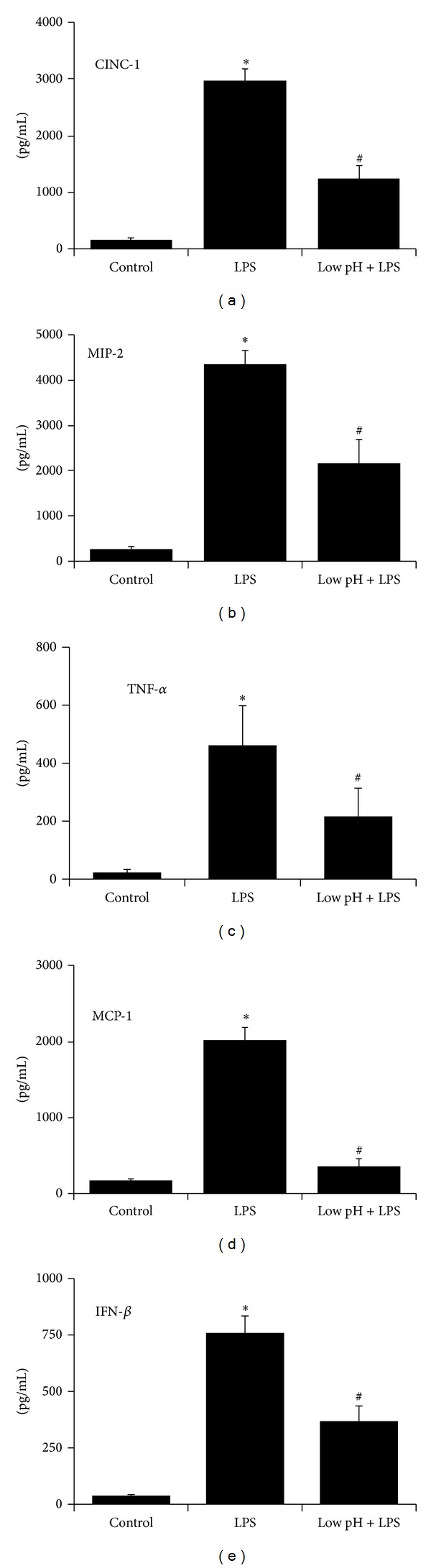
Effects of low pH stress on LPS-stimulated cytokine production in rat alveolar macrophages (aMØs). Isolated aMØs were stimulated *in vitro* with either saline (control), LPS alone (1.5 *μ*g/mL for 24 hrs), or LPS in combination with a preceding transient low pH stress injury (pH 1.75 for 1 min, Low pH + LPS). Cytokines produced were assessed by ELISA (CINC-1, MIP-2, MCP-1, and INF-*β*) or cytotoxicity bioassay (TNF-*α*). *P < 0.05 LPS versus control, ^#^ *P* < 0.05 LPS versus Low pH + LPS.

Bacterial LPS is an important structural lipoprotein found in Gram-negative bacteria that acts as a major activator of the antibacterial innate immune response. LPS ligates TLR4 to initiate intracellular signaling that results in production of antibacterial proinflammatory cytokines [12, 13]. Isolated rat aMØs were stimulated with LPS (1.5 *μ*g/mL for 24 hrs), and production of CINC-1, MIP-2, TNF-*α*, and MCP-1 was quantified (Figures 1(a)–1(d)). LPS significantly increased the production of CINC-1 from control levels of 153 ± 43 to 2944 ± 224 pg/mL (*P* < 0.001), MIP-2 from 250 ± 66 to 4347 ± 319 pg/mL (*P* < 0.001), TNF-*α* from 23 ± 10 to 462 ± 136 pg/mL (*P* = 0.01), and MCP-1 from 160 ± 28 to 2009 ± 172 pg/mL (*P* < 0.001). 

To investigate the effects of low pH stress on LPS-stimulated cytokine profile, LPS-treated aMØs were first exposed to low pH stress (acidified saline at pH 1.75 for 1 min). This regimen of transient low pH exposure was specifically determined to allow maximal acid exposure without decreasing cell viability (see Section 2). This exposure protocol also mimics the transient gastric acid exposure that is rapidly neutralized *in vivo*. Following low pH stress, LPS-induced cytokine release was significantly blunted compared with LPS alone (Figure 1); CINC-1 was reduced by 58% (*P* < 0.001), MIP-2 by 50% (*P* < 0.005), TNF-*α* by 53% (*P* < 0.05), and MCP-1 by 83% (*P* < 0.001).

LPS ligation of TLR4 signals two distinct cytosolic pathways, the MyD88 pathway and the TRIF (MyD88 independent) pathway. MyD88 signaling increases production of CINC-1, MIP-2, TNF-*α*, and MCP-1 through activation of the transcription factor, NF-*κ*B, while TRIF signaling increases IFN-*β* production through activation of interferon regulatory transcription factor 3 (IRF3). To evaluate the involvement of the TRIF pathway, aMØs were stimulated with LPS for 24 hrs, and the release of IFN-*β* was quantified using ELISA (see Section 2). LPS stimulation resulted in a significant increase in IFN-*β* production from control levels of 37.8 ± 5.8 pg/mL to 758.7 ± 75.4 pg/mL (*P* < 0.001, Figure 1(e)). Exposure to low pH stress reduced LPS-induced IFN-*β* production by 52% (*P* < 0.01). These findings suggest that low pH stress inhibits LPS-induced cytokine release through inhibition of both MyD88 and TRIF pathways.

### 3.2. Effects of Low pH Stress on LTA-Induced Cytokine Production in Alveolar Macrophages

**Figure 2:**
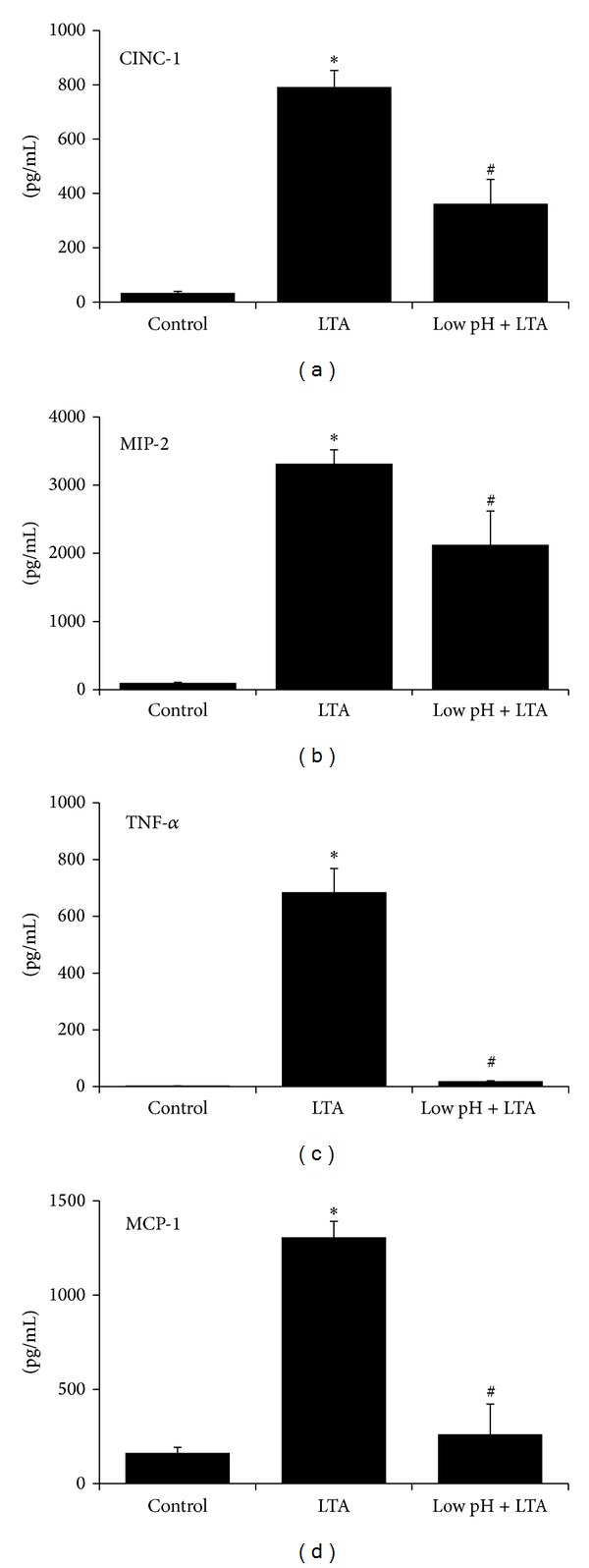
Effects of low pH stress on LTA-stimulated cytokine production in rat aMØs. Isolated aMØs were stimulated *in vitro* with either saline (control), LTA alone (30 mg/mL for 24 hrs), or LTA in combination with a preceding transient low pH stress injury (pH 1.75 for 1 min, Low pH + LTA). Cytokines produced were assessed by ELISA (CINC-1, MIP-2, and MCP-1) or cytotoxicity bioassay (TNF-*α*). *P < 0.05 LTA versus control, ^#^ *P* < 0.05 LTA versus Low pH + LTA.

The PAMP, LTA, is a major component of the outer cell wall of Gram-positive bacteria, and it shares many similarities with LPS in its proinflammatory effects. Cellular recognition of LTA is mediated by TLR2 ligation, which initiates cytosolic signaling that results in production of antibacterial cytokines [14, 15]. Isolated aMØs were stimulated *in vitro* with LTA (30 mg/mL for 24 hrs), and production of CINC-1, MIP-2, TNF-*α*, and MCP-1 was quantified (Figure 2). LTA increased the production of CINC-1 from control levels of 33 ± 5 to 792 ± 61 pg/mL (*P* < 0.001), MIP-2 from 99 ± 9 to 3316 ± 200 pg/mL (*P* < 0.001), TNF-*α* from 0.3 ± 0.04 to 685 ± 83 pg/mL (*P* < 0.001), and MCP-1 from 162 ± 29 to 1307 ± 84 pg/mL (*P* < 0.001).

To examine low pH stress on LTA-stimulated cytokines, aMØs were exposed to low pH stress (acidified saline at pH 1.75 for 1 min) prior to stimulation with LTA. Following low pH stress, LTA-induced cytokine production was significantly decreased compared to LTA alone (Figure 2); CINC-1 was reduced by 54% (*P* < 0.05), MIP-2 by 35% (*P* = 0.02), TNF-*α* by 97% (*P* = 0.02), and MCP-1 by 80% (*P* < 0.001). 

### 3.3. Role of Intracellular Ca^2+^ in Mediating Effects of Low pH Stress on LPS-Stimulated Responses

 Intracellular Ca^2+^ concentration ([Ca^2+^]_i_) is an important regulator of macrophage function [16–18]. To assess the role of [Ca^2+^]_i_ in mediating the effects of low pH stress on LPS-mediated responses, changes in [Ca^2+^]_i_ were determined using the Ca^2+^-sensitive fluorescent dye, Fura-2 AM (see Section 2). AMØs were exposed transiently to low pH stress and subsequently stimulated with LPS for 24 hrs. Fluorescence intensities (*R*_340/380_) were measured at different time intervals within this 24 hr period to identify variations in the temporal Ca^2+^ levels: immediate postexposure to LPS, after 30 min, 1 hr, 4 hrs, and 24 hrs of LPS exposure.

**Figure 3:**
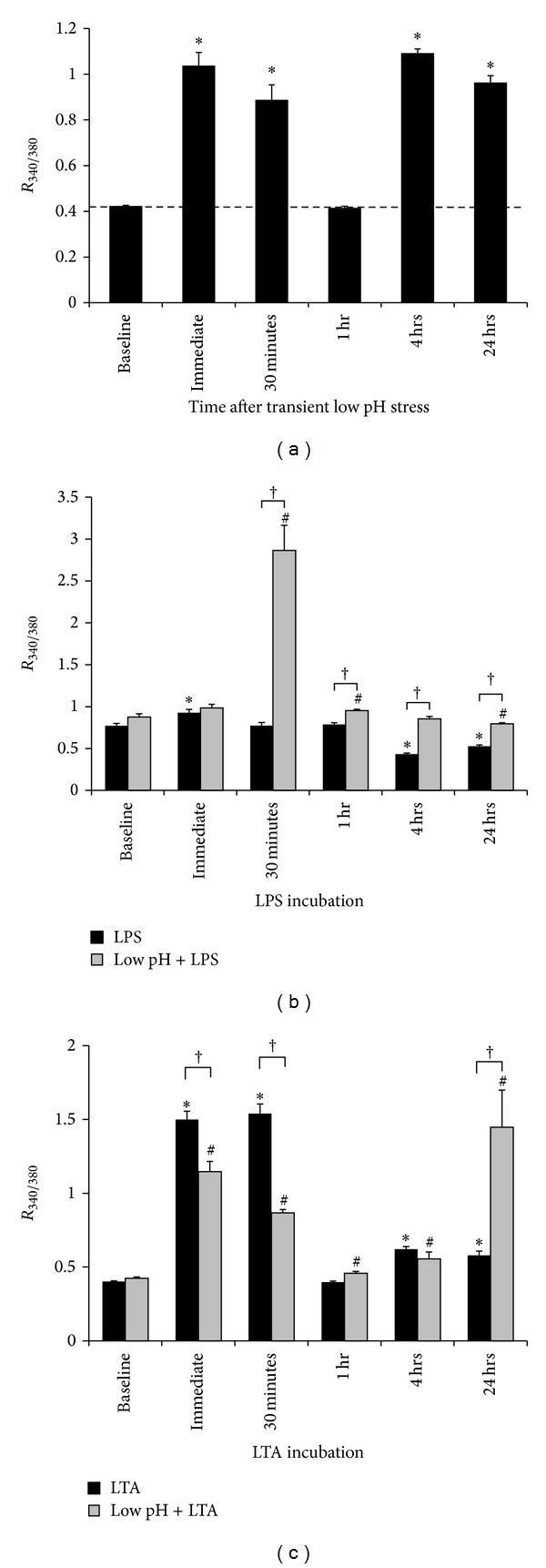
Intracellular Ca^2+^ concentration profile under various conditions, expressed as fluorescence excitation ratios (*R*_340/380_, proportional to [Ca^2+^]_i_) in Fura-2 AM-loaded aMØs. (a) Isolated aMØs loaded with Fura-2 AM were exposed to low pH stress *in vitro* and fluorescence ratios recorded at the indicated time points following low pH injury. (b) Fura-2 AM-loaded aMØs were stimulated with either LPS alone (black bar) or LPS preceded by transient low pH stress injury (Low pH + LPS, gray bar). (c) Fura-2 AM-loaded aMØs were stimulated with either LTA alone (black bar) or LTA stimulation preceded by a transient low pH stress injury (Low pH + LTA, gray bar). ^∗/#^ *P* < 0.05 versus corresponding baseline, ^†^ *P* < 0.05 presence of low pH stress versus absence of low pH stress.

First, changes in [Ca^2+^]_i_ due to low pH stress alone were assessed. Low pH stress increased *R*_340/380_, correlating with increased [Ca^2+^]_i_, in a biphasic pattern (Figure 3(a)), increasing from a baseline value of 0.42 ± 0.01 to 1.04 ± 0.06, *P* < 0.001. This increase in [Ca^2+^]_i_ persisted to the 30 min time point. At 1 hr after low-pH exposure, the ratio dropped to baseline levels (*R*_340/380_ = 0.42  ±  0.01, *P* = ns). This was followed by another rise in *R*_340/380_ at 4 hrs and 24 hrs after exposure (*R*_340/380_ of 1.09  ±  0.03, *P* < 0.001 and 0.96  ±  0.05, *P* < 0.001, resp.). 

We then examined the effects of LPS alone on [Ca^2+^]_i_. Changes in [Ca^2+^]_i_ were assessed at increasing duration of LPS incubation as described earlier. *R*_340/380_ increased immediately following LPS stimulation from control levels of 0.77 ± 0.01 to 1.04 ± 0.06 (*P* = 0.02) (Figure 3(b)). The elevation was short-lived with return to baseline levels at both 30 min and 1 hr. This was followed by low levels of [Ca^2+^]_i_ at 4 hrs and 24 hrs.

The effect of low pH stress on the LPS-mediated [Ca^2+^]_i_ profile was then investigated. AMØs exposed to a transient low pH stress prior to LPS stimulation resulted in increased [Ca^2+^]_i_ at 30 min after LPS exposure (Figure 3(b)), 0.89  ±  0.04 for LPS alone versus 2.86  ±  0.30 for low pH/LPS (*P* < 0.001). The increased *R*_340/380_ persisted at the 1 hr (*P* < 0.001), 4 hrs (*P* < 0.001), and 24 hrs LPS exposure times (*P* < 0.001).

### 3.4. Role of Intracellular Ca^2+^ in Mediating Effects of Low pH Stress on LTA-Stimulated Responses

To examine the role of [Ca^2+^]_i_ in mediating the effects of low pH stress on LTA-mediated responses, we identified changes in [Ca^2+^]_i_ in response to LTA alone and following combined low pH stress and LTA stimulation (Figure 3(c)). In Fura-2 AM-loaded aMØs, LTA stimulation significantly increased *R*_340/380_ from baseline levels of 0.40 ± 0.00 to 1.50 ± 0.06 (*P* < 0.001). This elevation persisted at 30 min and returned to baseline levels at 1 hr (*R*_340/380_ = 0.40 ± 0.01, *P* = ns). After an initial return to baseline levels, the *R*_340/380_ ratio developed a second wave of increased [Ca^2+^]_i_ at 4 hrs and 24 hrs, 0.62 ± 0.02 (*P* < 0.001 versus baseline) and 0.58 ± 0.03 (*P* < 0.001), respectively. 

The effect of low pH stress on LTA-induced Ca^2+^ profile was then investigated. LTA-stimulated aMØs were exposed to low pH conditions and changes in *R*_340/380_ monitored. There was immediate suppression by low pH stress on the LTA-induced [Ca^2+^]_i_ increase, 1.50 ± 0.06 (LTA) versus 1.15 ± 0.07 (LTA/Low pH), *P* < 0.01, and the [Ca^2+^]_i_ suppression persisted at 30 min after LTA exposure. At 1 hr and 4 hrs, there was no difference in the LTA-stimulated response, with or without low pH stress. At 24 hrs, low pH stress resulted in a robust increase in *R*_340/380_, 0.58  ±  0.03 (LTA) versus 1.45  ±  0.25 (LTA/Low pH), *P* < 0.001. 

These findings indicate that complex changes in [Ca^2+^]_i_ are induced by LPS and LTA, and the effect of low pH stress on [Ca^2+^]_i_ appears unique to the specific ligand/TLR interaction. In LPS-stimulated cells, low pH stress resulted in low level augmentation of [Ca^2+^]_i_ while, in LTA-stimulated cells, low pH stress initially suppressed the [Ca^2+^]_i_ increase but was followed by a robust augmentation in [Ca^2+^]_i_.

### 3.5. Effects of Intracellular Ca^2+^ Sequestration on the Modulatory Role of Low pH Stress on LPS-Stimulated Cytokine Production

**Figure 4:**
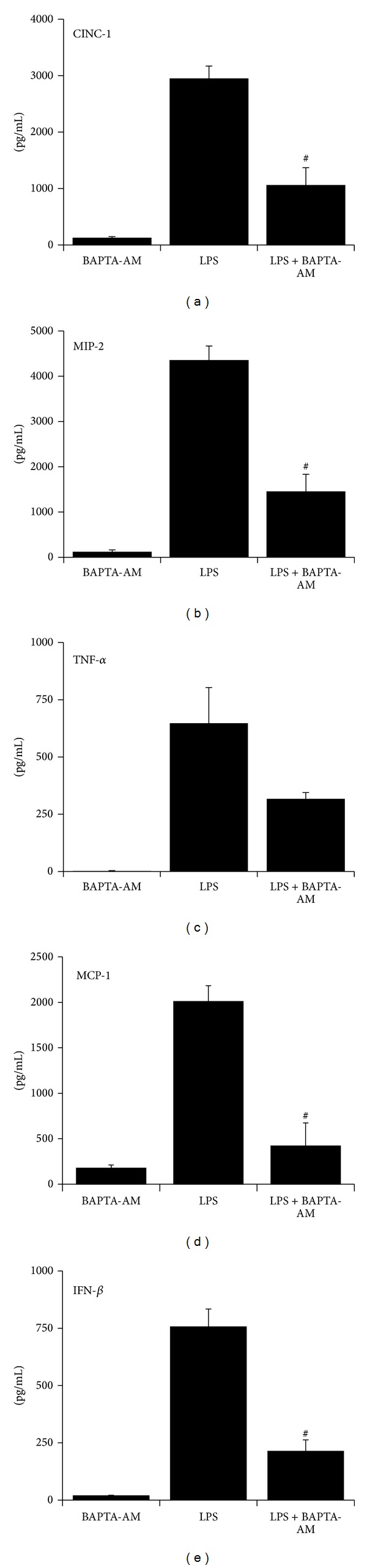
Effects of intracellular Ca^2+^ depletion (BAPTA-AM pretreatment) on LPS-stimulated cytokine production in aMØs. Isolated aMØs were stimulated *in vitro* with BAPTA-AM (5 *μ*M for 30 min), LPS (1.5 g/mL for 24 hrs), or LPS in combination with preceding BAPTA-AM (LPS + BAPTA-AM). Cytokines produced were assessed by ELISA (CINC-1, MIP-2, MCP-1, and INF-*β*) or cytotoxicity bioassay (TNF-*α*). ^#^ *P* < 0.05 LPS versus LPS + BAPTA AM.

To further investigate the role of Ca^2+^ in LPS-stimulated aMØs, BAPTA-AM was utilized as a cell-permeable Ca^2+^ chelator to effectively sequester [Ca^2+^]_i_ [19]. The general hypothesis is that if elevated [Ca^2+^]_i_ is necessary as an intermediate signal for LPS responses, BAPTA-AM will effectively inhibit these responses. AMØs were stimulated with LPS in the presence or absence of BAPTA-AM and cytokine production quantified. BAPTA-AM alone (control) did not result in any significant change in measured cytokines (Figure 4). In LPS stimulated cells, BAPTA-AM pretreatment decreased CINC-1 production by 64% (*P* < 0.001), MIP-2 by 67% (*P* < 0.001), MCP-1 by 79% (*P* < 0.001), and IFN-*β* (*P* < 0.001) from their LPS treatment levels. TNF-*α* levels trended down but were not statistically significant (*P* = 0.07).

**Figure 5:**
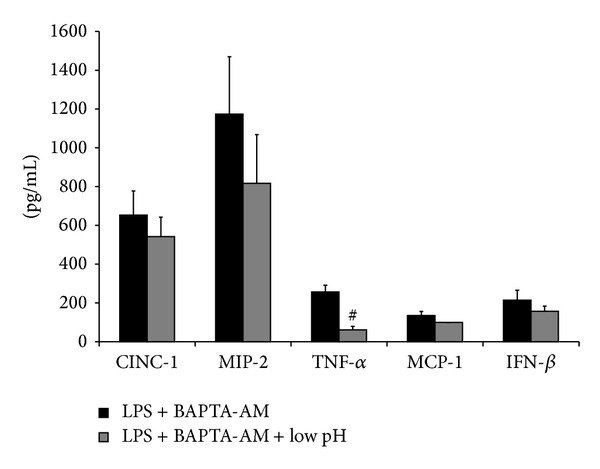
Effects of low pH stress on LPS-stimulated cytokine production in Ca^2+^-depleted (BAPTA-AM pretreatment) aMØs. Isolated aMØs were exposed to LPS and BAPTA-AM alone (LPS + BAPTA-AM) or in combination with low pH stress (LPS + BAPTA-AM + pH). Cytokines produced were assessed by ELISA (CINC-1, MIP-2, MCP-1, and INF-*β*) or cytotoxicity bioassay (TNF-*α*). ^#^ *P* < 0.05 LPS + BAPTA-AM versus LPS + BAPTA-AM + pH.

It was subsequently tested whether low pH stress can further modulate cytokine production in Ca^2+^-chelated, LPS-stimulated aMØs (Figure 5). Low pH stress did not affect CINC-1, MIP-2, MCP-1, and IFN-*β* production in LPS/BAPTA-AM-treated macrophages but significantly reduced TNF-*α* production by 82% (*P* < 0.001). 

### 3.6. Effects of Intracellular Ca^2+^ Sequestration on LTA-Mediated Cytokine Responses

**Figure 6:**
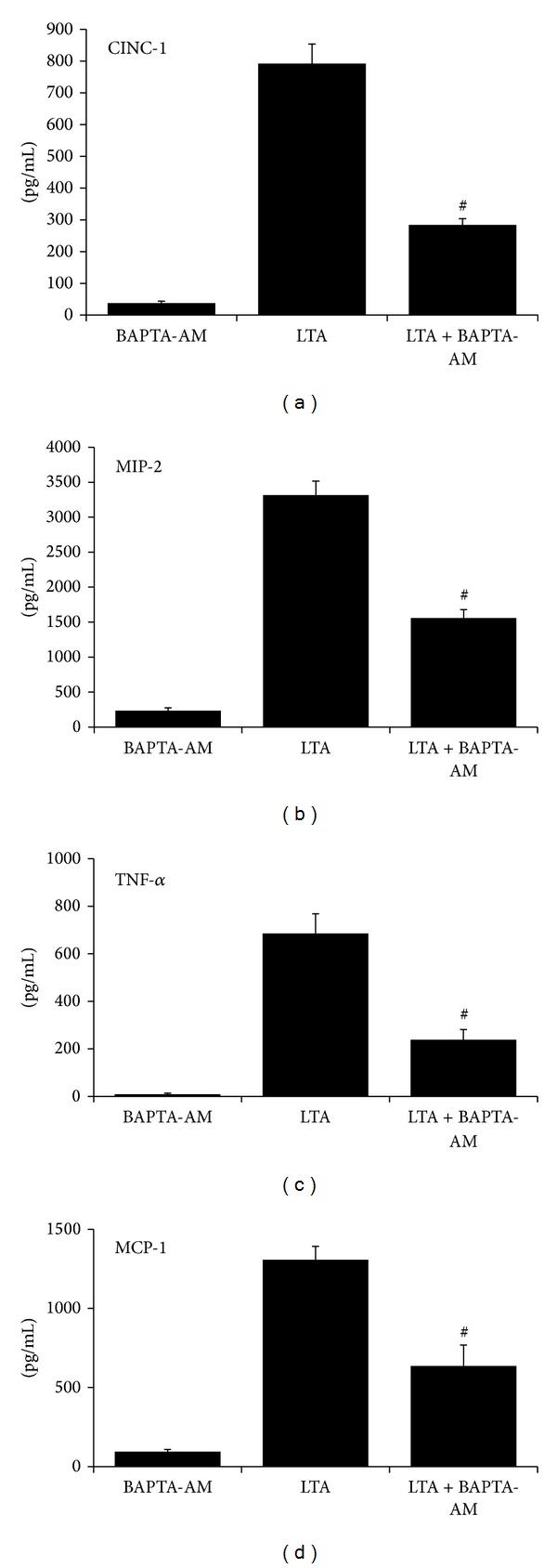
Effects of intracellular Ca^2+^ depletion (BAPTA-AM pretreatment) on LTA-stimulated cytokine production in aMØs. Isolated aMØs were stimulated *in vitro* with BAPTA-AM (5 *μ*M for 30 min), LTA (30 mg/mL for 24 hrs), or LTA in combination with preceding BAPTA-AM treatment (LTA + BAPTA-AM). Cytokines produced were assessed by ELISA (CINC-1, MIP-2, and MCP-1) or cytotoxicity bioassay (TNF-*α*). ^#^ *P* < 0.05 LTA versus LTA + BAPTA-AM.

**Figure 7:**
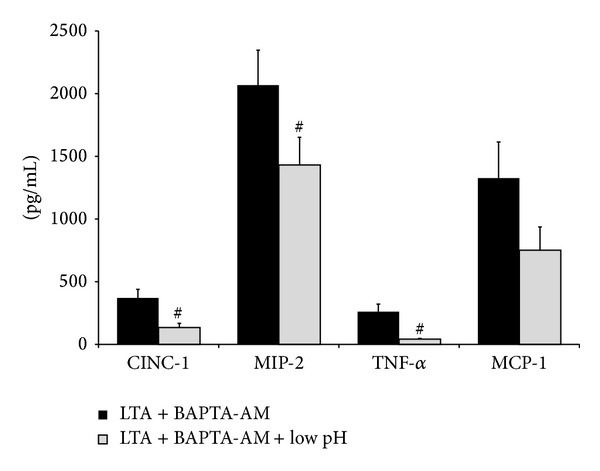
Effects of low pH stress on LTA-stimulated cytokine production in Ca^2+^-depleted alveolar macrophages (BAPTA-AM pretreatment). Isolated aMØs were exposed to LTA and BAPTA-AM alone (LTA + BAPTA-AM) or in combination with low pH stress (LTA + BAPTA-AM + pH). Cytokines produced were assessed by ELISA (CINC-1, MIP-2, and MCP-1) or cytotoxicity bioassay (TNF-*α*). ^#^ *P* < 0.05 LTA + BAPTA-AM versus LTA + BAPTA-AM + pH.

A set of experiments were performed using LTA-stimulated aMØs with BAPTA-AM. BAPTA-AM, in LTA-stimulated macrophages (Figure 6), reduced levels of CINC-1 by 64% (*P* < 0.001), MIP-2 by 53% (*P* < 0.01), TNF-*α* by 65% (*P* < 0.05), and MCP-1 by 51% (*P* < 0.05), when compared with LTA treatment in the absence of BAPTA-AM. It was subsequently investigated whether low pH stress would further modulate cytokine production in Ca^2+^-chelated, LTA-stimulated aMØs (Figure 7). Low pH stress suppressed levels of CINC-1 by 43% (*P* < 0.001), MIP-2 by 46% (*P* < 0.01), and TNF-*α* by 82% (*P* < 0.001) in LTA/BAPTA-AM-treated cells. Low pH stress tended to suppress MCP-1 (*P* = 0.06). 

### 3.7. Effects of Low pH Stress on Bacterial Clearance *In Vivo*

To determine the effects of low pH stress on bacterial clearance *in vivo*, mice were exposed to an acidic pulmonary aspiration injury followed by intratracheal instillation of either *E. coli* or *S. pneumoniae* (mimicking LPS and LTA response, resp.) (see Section 2). In animals inoculated with *E. coli*, starting inoculum was 5.3 × 10^6^ ± 0.5 × 10^6^ cfu (*n* = 17). Twenty-four hours after intratracheal *E. coli* challenge, resulting lung bacterial loads in the absence of low pH injury were 5.3 × 10^3^ ± 4.4 × 10^3^ cfu (*n* = 13) versus 1.1 × 10^5^ ± 0.3 × 10^5^ cfu (*n* = 17) in the presence of low pH injury, demonstrating an increased *E. coli* load after low pH injury. Estimated *E. coli* clearance (see bacterial clearance estimation under Methods section) was 3.7 ± 0.2 versus 1.9 ± 0.1, respectively (*P* < 0.001). In animals inoculated with *S. pneumoniae*, starting inoculum was 4.5 × 10^5^ ± 0 (*n* = 5). Twenty-four hours after intratracheal *S. pneumoniae* challenge, resulting lung bacterial loads in the absence of low pH injury were 5.8 × 10^3^ ± 2.9 × 10^3^ cfu versus 8.4 × 10^7^ ± 3.1 × 10^7^ cfu in the presence of low pH injury, demonstrating a profound increase in total *S. pneumoniae* load, in excess of the starting inoculum. Estimated *S. pneumoniae* clearance was 2.4 ± 0.4 versus −2.19 ± 0.2, respectively (*P* < 0.001). 

## 4. Discussion

The innate immune system recognizes a wide variety of bacterial pathogens through a series of PRRs including TLRs, C-type lectin, and NOD-like receptors. The TLRs are the most widely studied PRRs with at least 10 recognized subtypes, each recognizing specific microbial patterns. In this study we examined TLR4- and TLR2-cytosolic signal transduction pathways through the use of LPS and LTA, respectively, to identify detrimental effects of nonlethal low pH stress on aMØ function. Clinical consequences of gastric aspiration are well established, ranging from a self-limiting aspiration pneumonitis to life threatening lung injury as seen in ARDS. Superimposed bacterial pneumonia is a frequent and deleterious sequela of gastric aspiration. *In vivo* animal studies have demonstrated that gastric acid aspiration results in a severe heterogeneous lung injury [20] which involves both direct low pH stress and indirect, non-pH stress-induced inflammation. This inflammatory response involves mechanisms that include direct tissue damage [11], impairment of surfactant function [20], and augmented recruitment of inflammatory/immune cells [11].

aMØs are sentinel cells that act as the initial line of defense against bacterial infection and are exposed to the inhaled fluid during a gastric aspirate event. This fluid often includes a low pH component, partially digested food particles, and potentially different degrees of bacterial loading arising from oropharyngeal and/or gastric colonization. The role of low pH stress in modulating aMØs function contributing to susceptibility to bacterial infection is not completely understood. Our group used an *in vitro* model to mimic low pH stress exposure of aMØs and identify direct changes in LPS- and LTA-mediated cytokine production.

**Figure 8:**
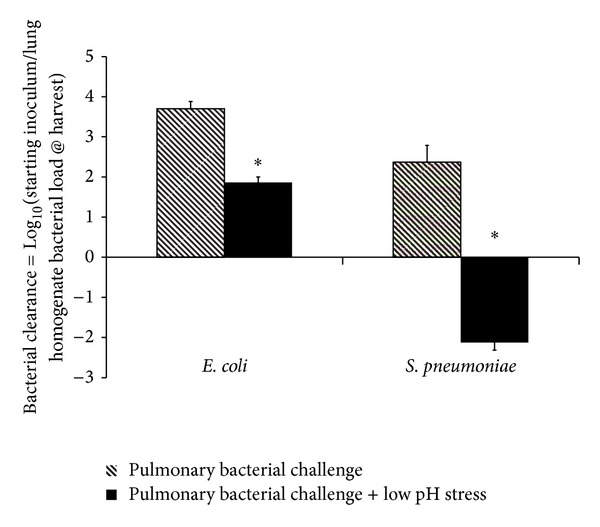
Effect of acid aspiration (low pH stress) on bacterial clearance *in vivo* was assessed using a mouse model of pulmonary bacterial infection. Pulmonary bacterial challenge was initiated using intratracheal administration of known titers (starting inoculum) of *E. coli* (left panel) or *S. pneumoniae* (right panel). Acid aspiration was modelled using intratracheal administration of acidified saline (pH 1.25) prior to bacterial challenge (pulmonary bacterial challenge + low pH stress). Lung homogenates were prepared 24 hr after intratracheal bacterial challenge and bacterial load quantified. *P < 0.05 Bacterial pulmonary challenge alone versus bacterial pulmonary challenge + low pH stress. *P < 0.05 versus bacterial inoculation alone.

Our primary findings demonstrated dramatic inhibition of proinflammatory cytokine secretion (i.e., CINC-1, MIP-2, TNF-*α*, and MCP-1) by aMØs exposed to a low pH stress. The model used in our experiment was specifically designed to achieve a level of acid exposure to induce the highest level of low pH stress without a decrease in cell viability. Our preliminary work on establishing cell integrity ensured that the reduced cytokine levels were not a result of changes in aMØs number but rather reflected a true alteration in aMØs signaling. The profound reduction in cytokine levels induced by low pH stress would be anticipated to negatively impact the aMØs sentinel response to bacterial infection. Our *in vivo* animal data supports this hypothesis demonstrating suppression of both *E. coli* and *S. pneumoniae* bacterial clearance (Figure 8).

As an early surveillance mechanism to detect infection, TLRs recognize specific microbial components or PAMPs. TLR2, which is activated by LTA, is a heterodimer in conjunction with TLR1 and TLR6 [21–23]. Other moieties reported to be recognized by TLR2 are lipoproteins, mycobacterial lipoarabinomannan [24], and rare lipopolysaccharide species [25]. On the other hand, TLR4, which principally acts as a homodimer, is important for recognition of LPS [22]. TLR4 has also been reported to recognize viral proteins [26, 27] and several damage-associated molecular patterns (DAMPs) which are products of tissue injury. DAMPs include ligands such as HMGB1, calgranulins, heat shock protein B8 [28], fibrinogen [29], and breakdown products of heparan sulfate polysaccharides [30]. Four adaptor proteins mediate TLRs signaling, and their differential recruitment partially affords pathogen response specificity; the adaptor proteins include MyD88, TRIF (toll-receptor-associated activator of interferon), MAL (MyD88 adapter-like), and TRAM (TRIF-related adapter molecule) [31]. Both TLR2 and TLR4 utilize MyD88 and are the prototypic pathway for TLRs signaling. Recruitment of MyD88 initiates a cascade involving sequential recruitment/activation of IRAK4 (IL-1R-associated kinase 4), IRAK1, tumor-necrosis-factor-receptor-associated factor 6 (TRAF6), and TAK1/TAB1/2/3. Ultimately, three important, well-characterized pathways are activated: nuclear factor-(NF-*κ*B) [32], mitogen-activated protein kinases (MAP) [33], and phosphoinositide 3-kinases (PI3K) [34]. These distinct pathways result in induction of transcription, mRNA stabilization, and translation of a wide variety of proinflammatory cytokines and chemokines important for bactericidal activity and chemotaxis/recruitment of other immune competent cells [35–37]. TLR4, however, in addition to utilizing MyD88 pathway also utilizes MyD88-independent pathway, recruiting the adaptor protein TRIF/TRAM, leading to activation of interferon regulatory factor 3 (IRF3) and resulting in induction of IFN-*β* [38]. Consistent with previously reported data, our results demonstrated a robust LTA- and LPS-mediated induction of proinflammatory cytokines. Exposure to low pH stress dramatically inhibited LPS- and LTA-mediated CINC-1, MIP-2, TNF-*α*, and MCP-1 levels (Figures 1 and 2), as well as a parallel inhibition of LPS-mediated IFN-*β* levels (Figure 1(e)). Although the exact mechanism is unclear, this suggests that low pH stress may act upstream in the intracellular signaling involved in the MyD88 and TRIF pathways, possibly involving receptor binding and/or initial adaptor protein association.

Cytosolic Ca^2+^ is a ubiquitous second messenger important in many intracellular processes including induction of key transcriptional processes [39]. TLR2 and TLR4 ligand stimulation, in part, utilizes Ca^2+^ to generate increased cytokine production. The mechanism of Ca^2+^ flux continues to be investigated, but a mechanism that has been suggested involves receptor ligand binding causing receptor phosphorylation by c-Src [40, 41]. This leads to activation of PI3K and phospholipase C*γ*2 (PLC*γ*2), affecting release of intracellular Ca^2+^ store through IP3R activation in the endoplasmic reticulum [41]. The exact Ca^2+^-dependent step affecting NF-*κ*B activity is still unclear, but several potential candidates may include traditional Ca^2+^-sensitive protein kinases such as protein kinase C*α* (PKC*α*), PKC*β*, PKC*γ*, and calmodulin-dependent kinases that have been shown to modulate NF-*κ*B activity [42, 43]. Cytosolic changes in [Ca^2+^]_i_ are also required for optimal TLR4 trafficking to the endosomes and the phosphorylation/nuclear localization of IRF3 [44]. Our data further confirms the essential role of Ca^2+^ in TLR2 and TLR4 signaling, through demonstration of [Ca^2+^]_i_ increases in response to LPS and LTA (Figure 3) and reduction in cytokine levels following intracellular Ca^2+^ depletion (Figures 4–6). By tracking the changes in [Ca^2+^]_i_ at different time points, we demonstrated the complex nature of the cytosolic Ca^2+^ profile generated by LPS with an early transient single peak followed by low level [Ca^2+^]_i_ (Figure 3(b)). LTA stimulation, on the other hand, resulted in a biphasic pattern with an early peak followed by return to baseline and a secondary peak at a later time point (Figure 3(c)). Overall LPS, in contrast to LTA, resulted in a less robust Ca^2+^ response. The nature of this difference is unclear; however, this short-lived but biologically important Ca^2+^ response to LPS is consistent with other reported literature on LPS-stimulated Ca^2+^ response, demonstrating only transient increases in Ca^2+^, predominantly in the range of 50–150 nM [45–47]. Combined exposures of LPS or LTA with low pH stress did not result in a simple summation of Ca^2+^ profile but modified LPS- and LTA-induced Ca^2+^ profiles in distinct patterns. In LPS-stimulated cells, low pH stress tended to generally increase [Ca^2+^]_i_ (Figure 3(b)). In contrast, low pH stress suppressed [Ca^2+^]_i_ in the early phase of LTA stimulation but augmented it at a later phase (Figure 3(c)). 

To further investigate the role of Ca^2+^, we also utilized BAPTA-AM to effectively sequester free intracellular Ca^2+^. BAPTA-AM at the concentration used in our experiments has been used by different laboratories as a Ca^2+^-selective sequestrant [19, 48–50]. Although other non-Ca^2+^-dependent mechanisms can potentially be altered by BAPTA-AM, it appears to have a very high selectivity over other bivalent cations, and its biologic activities have been linked predominantly to its ability to sequester Ca^2+^ [19, 48–50]. Under a [Ca^2+^]_i_-clamped state (BAPTA-treated cells), low pH stress failed to modify LPS-mediated production of proinflammatory cytokines (Figure 5) while continuing to suppress LTA-mediated cytokine production (Figure 7). This differential response suggests that the interaction of low pH stress on LPS/TLR4 and LTA/TLR2 pathways may be distinct with TLR4 involving predominantly Ca^2+^-dependent mechanisms, while that of TLR2 may involve Ca^2+^-independent mechanisms.

In summary, our findings demonstrate a strong suppressive direct effect of low pH stress on the ability of aMØs to mount an adequate antibacterial proinflammatory response following TLR2 and TLR4 activation. The site of action of the low pH stress appears to involve both the MyD88 and TRIF pathways. Ca^2+^ is an essential component for efficient elaboration of LPS- and LTA-mediated cytokine response but plays a differential role in mediating the suppressive effects of low pH stress. Changes in [Ca^2+^]_i_ appear to be required for LPS-stimulated responses but are less important for the LTA-mediated response. The mechanism for this differential role of cytosolic Ca^2+^ signaling needs further investigation, but the data presented in this study suggest that the mechanistic effect of low pH stress cannot be generalized across the various TLRs signaling cascades. These findings further our understanding of aMØs dysfunction following acute gastric aspiration and the role of this resident sentinel lung cell in the increased susceptibility to bacterial infection.
